# Comparison of thermodilution measured extravascular lung water with chest radiographic assessment of pulmonary oedema in patients with acute lung injury

**DOI:** 10.1186/2110-5820-3-25

**Published:** 2013-08-11

**Authors:** Lisa M Brown, Carolyn S Calfee, James P Howard, Thelma R Craig, Michael A Matthay, Daniel F McAuley

**Affiliations:** 1 Department of Surgery, Division of Cardiothoracic Surgery, Washington University, St. Louis, MO, USA; 2 Cardiovascular Research Institute, University of California, San Francisco, CA, USA; 3 Department of Medicine, Division of Pulmonary and Critical Care, University of California, San Francisco, CA, USA; 4 Department of Anesthesia, University of California, San Francisco, CA, USA; 5 Department of Pediatrics, Division of Critical Care Medicine, University of California, San Francisco, CA, USA; 6 Regional Intensive Care Unit, Royal Victoria Hospital, Belfast, UK; 7 Centre for Infection and Immunity, The Queen’s University of Belfast, Belfast, UK; 8 University of California, 505 Parnassus Avenue, Room M-917, San Francisco, CA 94143-0624, USA

**Keywords:** Extravascular lung water, Chest radiograph, Acute lung injury, Acute respiratory distress syndrome

## Abstract

**Background:**

Acute lung injury and the acute respiratory distress syndrome (ALI/ARDS) are characterized by pulmonary oedema, measured as extravascular lung water (EVLW). The chest radiograph (CXR) can potentially estimate the quantity of lung oedema while the transpulmonary thermodilution method measures the amount of EVLW. This study was designed to determine whether EVLW as estimated by a CXR score predicts EVLW measured by the thermodilution method and whether changes in EVLW by either approach predict mortality in ALI/ARDS.

**Methods:**

Clinical data were collected within 48 hours of ALI/ARDS diagnosis and daily up to 14 days on 59 patients with ALI/ARDS. Two clinicians scored each CXR for the degree of pulmonary oedema, using a validated method. EVLW indexed to body weight was measured using the single indicator transpulmonary thermodilution technique.

**Results:**

The CXR score had a modest, positive correlation with the EVLWI measurements (r = 0.35, *p* < 0.001). There was a 1.6 ml/kg increase in EVLWI per 10-point increase in the CXR score (*p* < 0.001, 95% confidence interval 0.92-2.35). The sensitivity of a high CXR score for predicting a high EVLWI was 93%; similarly the negative predictive value was high at 94%; the specificity (51%) and positive predictive value (50%) were lower. The CXR scores did not predict mortality but the EVLW thermodilution did predict mortality.

**Conclusion:**

EVLW measured by CXR was modestly correlated with thermodilution measured EVLW. Unlike CXR findings, transpulmonary thermodilution EVLWI measurements over time predicted mortality in patients with ALI/ARDS.

## Background

Acute lung injury and the acute respiratory distress syndrome (ALI/ARDS) are characterized by an increase in pulmonary capillary permeability to protein leading to extravasation of protein-rich oedema fluid, known as extravascular lung water (EVLW), into the alveoli [1,2]. Importantly, the quantity of EVLW measured early in the course of ALI can predict survival [3,4] Therefore, quantitative and accurate measure of EVLW may be a valuable tool for assessment of this patient population. Furthermore, successive and reliable estimates of EVLW could be an effective guide to fluid management and diuretic therapy, potentially offering a superior approach to the contemporary clinical strategies used in patients with ALI/ARDS [5-7]. Therefore, methods to accurately quantify the amount of EVLW may be of value to clinicians who treat patients with ALI/ARDS.

The most commonly used method to estimate the amount of EVLW is the chest radiograph [8]. EVLW also may be measured at the bedside using transpulmonary thermodilution methods [9-11]. Thermodilution methods are invasive because they require that the patient have both a central venous and a femoral arterial catheter. It is unclear whether these two methods of evaluating EVLW provide similar information to the clinician. If the chest radiograph findings can accurately predict the amount of EVLW, then it may not be necessary to use invasive methods to measure EVLW. However, if not, measuring EVLW may contribute additional useful information in critically ill patients with ALI/ARDS.

Several early studies of critically ill patients with pulmonary oedema reported that serial chest radiographs were not useful for estimating absolute or changes in the EVLW [12]. These studies found a moderate positive correlation between the chest radiograph findings and the quantity of EVLW measured with either the gravimetric or transpulmonary thermodilution methods [12-17]. However, all of these studies were done more than 20 years ago, and since that time both the technology of portable chest radiographs and measurement of EVLW have improved [18,19]. Furthermore, using an objective chest radiograph scoring system may improve the ability to measure pulmonary oedema using chest radiograph findings [20]. Prior studies comparing the chest radiograph with EVLW measured using the thermodilution method used double indicator methods to measure the amount of EVLW [12,14-17]. Currently, the most common thermodilution method of measuring EVLW is the single-indicator transpulmonary thermodilution method (PiCCO, Pulsion Medical Systems, Munich, Germany) [9]. This method has been validated by comparison with the “gold standard” gravimetric method in experimental animal studies [21-23]. Several investigators have studied EVLW in critically ill patients [24-31]. There is some evidence that EVLW measured by the single-indicator transpulmonary thermodilution method early in the course of ALI/ARDS, particularly if indexed to predicted body weight, is associated with poor outcome [3,32].

The main purpose of this study was to determine whether a chest radiograph score that has been recently validated [33] could predict EVLW measured using the transpulmonary thermodilution method in patients with ALI/ARDS. We also determined whether changes in EVLW on consecutive ICU days as measured by the chest radiograph and the transpulmonary thermodilution method predict ventilator-free days and mortality in patients with ALI/ARDS.

## Methods

### Subjects

Patients for this study were enrolled in a randomized, clinical trial investigating the effects of simvastatin on EVLW in patients with ALI/ARDS [34]. Patients were eligible for enrollment if they met the American-European Consensus Conference criteria for ALI or ARDS [35]. The exclusion criteria included creatine kinase (CK) >10 times upper limit normal range, liver transaminases >3 times upper limit normal range, patients with severe renal impairment (calculated creatinine clearance <30 ml/min) not receiving renal replacement therapy, patients with severe liver disease (Child’s Pugh score >11), known lactose intolerance, current treatment with any lipid lowering agent including statins, contraindications to enteral drug administration; age <18 years; pregnancy; participation in a clinical trial with an investigational medicinal product within 30 days, unlikely to survive beyond 48 hours, and declined consent. Baseline EVLW in the first 44 patients from this cohort have been published elsewhere in a paper describing the relationship between baseline EVLW indexed to predicted body weight and mortality in ALI/ARDS [32]. Mechanically ventilated patients admitted to a 17-bed tertiary referral center intensive care unit (ICU) in Northern Ireland were prospectively screened for ALI/ARDS during a 2-year period. The local institution and ethics committee approved the protocol for the study, and all patients or their surrogate provided informed consent.

### Data collection

Clinical data were collected within 48 hours of diagnosis of ALI/ARDS and once per day for up to 14 consecutive days. The aetiology of ALI/ARDS was recorded, and patients with pneumonia and septic shock requiring vasopressor support were considered to have an infectious etiology. Baseline Acute Physiology and Chronic Health Evaluation Score (APACHE II), Simplified Acute Physiology Score (SAPS II), Sequential Organ Failure Assessment (SOFA), and Lung Injury Score (LIS) were determined for each patient.

The PaO_2_/FiO_2_ ratio was calculated using arterial blood gas samples and ventilator data. Ventilator parameters recorded included tidal volume, respiratory rate, minute ventilation, positive end expiratory pressure, mean airway pressure, peak pressure, plateau pressure, and static respiratory compliance.

### Chest radiograph score

A chest radiograph scoring system was used by a two clinician panel to score each chest radiograph who interpreted the chest radiographs together at the same time. Thus, separate interpretations were not done, so it was not possible to assess interobserver variability by a kappa value. The clinicians were blinded to the EVLWI measurements. First, the quality of the chest radiograph was classified as acceptable, borderline, or not usable. Next, the probability of atelectasis (yes/no) was determined in each of the following four quadrants: right upper (RU), right lower (RL), left upper (LU), left lower (LL). Finally, the degree of alveolar oedema in each of the four quadrants was scored as follows: 1) 0-25%, 2) 25-50%, 3) 50-75%, and 4) 75-100%. The middle value of each of the alveolar oedema scores was used to categorize each of the chest radiograph quadrants as follows: 1) 12.5%, 2) 37.5%, 3) 62.5%, and 4) 87.5%. The four alveolar oedema scores (RU, RL, LU, LL) for each chest radiograph were totaled and divided by four to generate an overall chest radiograph score. This scoring system has been recently validated [33]. Chest radiographs were taken daily between 9:00 and 10:00 a.m. using portable equipment (exposure factor 90 kV, 1.4 mAs, a digital system).

### EVLWI measurement

EVLW was measured within 48 hours of diagnosis of ALI/ ARDS and once per day between 8:00 and 9:00 a.m. for up to 14 consecutive days using the single-indicator transpulmonary thermodilution technique (PiCCO Pulsion Medical Systems, Munich, Germany). A 15-mL bolus of 0.9% normal saline at 4°C was injected into a central venous catheter. The change in temperature of this bolus was detected at the thermistor tip of the femoral arterial line and a thermodilution curve was generated. The EVLW was determined based on the characteristics of the thermodilution curve [9]. Three bolus injections were used to calculate the mean EVLW. The EVLW measurements were indexed to the patients’ predicted body weight (EVLWI). EVLWI was calculated by dividing the total EVLW by the predicted body weight. The predicted body weight (in kilograms) was calculated as 0.91 (height [cm] −152.4) + 50 for males or + 45.5 for females. The clinicians treating the patients had no knowledge of the results of the EVLWI measurements.

### Statistical analyses

The relationship between the chest radiograph score and EVLWI measurements was graphed using scatterplots and the correlation between the two was calculated using Spearman’s correlation. Locally weighted scatterplot smoothing (Lowess) was used to analyze the smoothed, nonparametric relationship between CXR score, and EVLWI. Linear regression was used to determine whether the chest radiograph score predicts EVLWI. The model was clustered by patient, in order to account for repeated measures.

We determined the sensitivity, specificity, positive predictive value, and negative predictive value of the chest radiograph scores using the transpulmonary thermodilution EVLWI measurements as the “gold standard.” The EVLWI measurements were split into two groups: high EVLWI (≥16 ml/kg) and low EVLWI (<16 ml/kg) based on prior data demonstrating that EVLWI ≥16 ml/kg predicted mortality with 100% specificity and 86% sensitivity [3,32]. The chest radiograph scores were also split into two groups: high score (>85) and low score (≤85) based on analysis of our data, in which one third of the scores were ≤85 and the other two thirds were >85.

Next, we compared how well the baseline chest radiograph score, EVLWI, and PaO_2_/FiO_2_ predicted mortality (within 30 days of hospital admission) and ventilator-free days. The *t* test was used to determine whether the baseline values of these three predictors differed between survivors and nonsurvivors. Univariate logistic regression was used to determine whether these three baseline values predicted ICU mortality. The area under the receiver operating characteristic curve was determined for each of these three predictors and compared. Univariate linear regression was used to determine whether these three baseline values predicted ventilator-free days.

Finally, Cox regression was performed using the chest radiograph score, EVLWI, and PaO_2_/FiO_2_ as time-dependent covariates to determine whether the change in each of these three variables predicts time to death. All patients were followed from the day of randomization until hospital discharge or death. A follow-up time to and including 30 days was used for this analysis. A *p* value < 0.05 was considered statistically significant. STATA SE version 10.1 (College Station, TX) was used for all statistical analyses, which were reviewed by a biostatistician.

## Results

There were 59 patients included in these analyses and 476 total observations. The baseline characteristics of the cohort are presented in Table 1. No patients were lost to follow-up.

**Table 1:** Baseline characteristics of the ALI/ARDS cohort (n = 59)

**Characteristic**	**n = 59**
Age	54 (38–64)
Male gender	45 (76%)
**Etiology of ALI/ARDS**	
Trauma	19 (32%)
Pneumonia	11 (19%)
Aspiration	9 (15%)
Sepsis	9 (15%)
Pancreatitis	2 (3%)
Other/Unknown	9 (15%)
Extravascular lung water index (ml/kg)	11 (9–17)
Chest radiograph score	87.5 (68.75–87.5)
APACHE II score	25 (19-30)
SAPS II score	53 (42-63)
SOFA score	10 (8-12)
Lung injury score	2.5 (2-3)
**Respiratory parameters**	
PaO_2_ / FiO_2_	170 (129–202)
Mean airway pressure	13 (9–16)
Peak airway pressure	22 (17–25)
Plateau pressure	22 (17–24)
Compliance	42 ± 15
Dead space fraction	0.58 (0.52–0.65)
Minute ventilation	9 (8–11)
Tidal volume	500 (427–560)
Respiratory rate	18 (15–20)
PEEP	8 (6–10)
**Cardiovascular parameters**	
Heart rate	94 ± 19
Systolic blood pressure	116 (104–127)
Mean arterial pressure	78 (70–84)
Central venous pressure	12 ± 4

Data are median (interquartile range), number (%), or mean ± standard deviation.

### Correlation between chest radiograph score and EVLWI measurements

**Figure 1:**
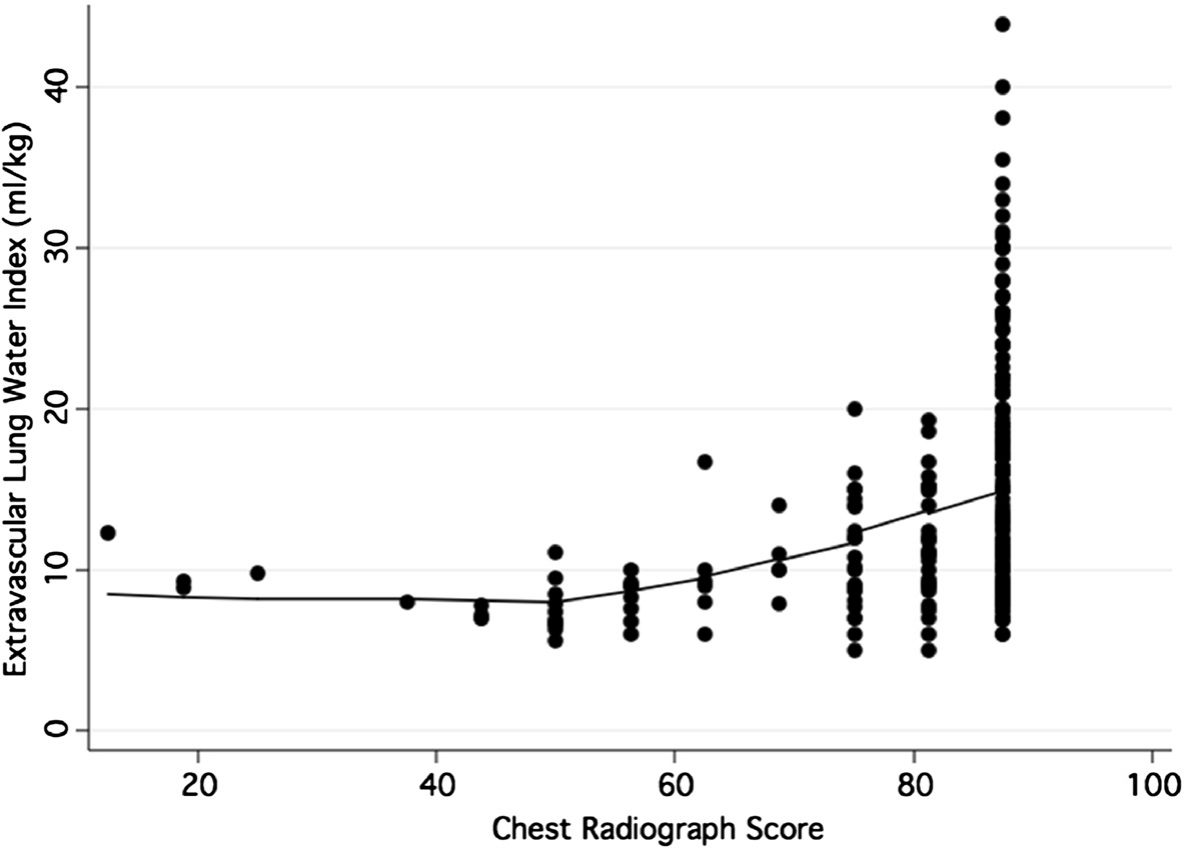
**Scatter plot of extravascular lung water index (EVLWI) versus chest radiograph score with a Lowess smoothing line.** EVLWI measurements and chest radiograph scores are from all days for which data are available (n = 476).

The chest radiograph scores were positively, but modestly, correlated with the EVLWI measurements (r = 0.35, *p* < 0.001; Figure 1). In an unadjusted linear model, higher values of the chest radiograph score were associated with higher values of EVLWI. Specifically, there was a 1.6 ml/kg increase in same day EVLWI per 10-point increase in chest radiograph score (*p* < 0.001, 95% confidence interval (CI) 0.92-2.35).

As we have done in a prior study [33], in order to investigate the potential confounding effect of atelectasis or suboptimal chest radiographs, we performed additional analyses, first excluding all observations with possible atelectasis in any lung quadrant (remaining, n = 352) and subsequently excluding all observations with poor radiograph quality or possible atelectasis (remaining, n = 261). In both sensitivity analyses, the correlations between the chest radiograph score and EVLWI measurements were stronger and there was a greater increase in EVLWI per increase in chest radiograph score (Table 2).

**Table 2:** Correlation between chest radiograph score and EVLWI measurements excluding chest radiographs of borderline quality or with possible atelectasis and of borderline quality

	**Possible atelectasis**	**Possible atelectasis**
	**excluded (n = 352)**	**and borderline quality**
		**excluded (n = 261)**
Chest radiograph score	r = 0.41, *p* < 0.001	r = 0.48, *p* < 0.001
correlation with the EVLWI		
Increase in EVLWI (ml/kg)^a^	1.8 *p* < 0.001,	2.0 *p* < 0.001,
	95% CI (0.98–2.64)	95% CI (1.1–2.81)

^a^Per 10–point increase in chest radiograph score.

The sensitivity of the chest radiograph score (cutoff >85 based on prior published studies [3,24]) as a diagnostic test for detecting EVLW compared with EVLWI as measured by the thermodilution method was high (93%) as was the negative predictive value (94%). However, the specificity (51%) and positive predictive values (50%) were lower.

### Chest radiograph score and EVLWI measurements and outcomes

Mortality was 31%. The baseline chest radiograph score and EVLWI were both higher in ICU nonsurvivors compared with survivors (Table 3). The baseline PaO_2_/FiO_2_ ratio was not quite statistically significant in nonsurvivors (Table 4). In logistic regression models, the baseline EVLWI and the PaO_2_/FiO_2_ predicted mortality, and there was a trend towards statistical significance for the ability of the baseline chest radiograph score to predict ICU mortality (Table 4). The areas under the receiver operating characteristic curves for each of the three models were similar. The baseline chest radiograph score and the PaO_2_/FiO_2_ ratio predicted ventilator free days (Table 5). The changes in daily thermodilution measured EVLWI measurements and PaO_2_/FiO_2_ ratio, unlike the chest radiograph score, were predictive of time to death (Table 6).

**Table 3:** **Baseline chest radiograph score, EVLWI, and PaO**_**2**_**/FiO**_**2 **_**in ICU survivors and nonsurvivors**

**Baseline predictor**	**Mean ± SD**	**95% CI**	***p *value**^**a**^
**Chest radiograph score**			
Survivors (n = 41)	74 ± 19	(68–80)	0.007
Nonsurvivors (n = 18)	84 ± 8	(79–88)	
**EVLWI**			
Survivors (n = 41)	12 ± 5	(11–14)	0.05
Nonsurvivors (n = 18)	17 ± 9	(12–21)	
**PaO**_**2**_**/FiO**_**2**_			
Survivors (n = 41)	179 ± 47	(164–194)	0.08
Nonsurvivors (n = 18)	146 ± 67	(110–181)	

^**a**^*p* value for the difference in baseline predictor between survivors and nonsurvivors, *t* test with unequal variance.

**Table 4:** Univariate logistic regression models for predicting ICU mortality using baseline predictors

**Predictor**^**a**^	**Unadjusted**	***p *value**	**95% CI**	**AUROC**^**b**^
	**odds ratio**			
Chest radiograph score	1.77	0.07	(0.95–3.3)	0.66
Extravascular lung	1.12	0.03	(1.01–1.24)	0.68
water index (ml/kg)				
PaO_2_ / FiO_2_	0.89	0.04	(0.79–0.99)	0.65

^a^Per 10–point increase in chest radiograph score and PaO_2_/FiO_2_.

^b^Area under the receiver operating characteristic curve.

No statistically significant difference between these three AUROCs, *p* = 0.9.

**Table 5:** Univariate linear regression models for predicting ventilator–free days using baseline predictors

**Predictor**^**a**^	**Unadjusted β**	***p *value**	**95% CI**
	**coefficient**		
Chest radiograph score	−1.75	0.007	(−2.99 to −0.51)
Extravascular lung water	−0.18	0.3	(−0.53 to 0.17)
index (ml/kg)			
PaO_2_ / FiO_2_	0.41	0.04	(0.02–0.81)

^a^Per 10–point increase in chest radiograph score and PaO_2_/FiO_2_.

**Table 6:** Cox regression models for predicting death

**Predictor**^**a**^	**Unadjusted**	***p *value**	**95% CI**
	**hazard ratio**		
Chest radiograph score	1.72	0.3	(0.59–4.99)
Extravascular lung water	1.14	0.001	(1.05–1.23)
index (ml/kg)			
PaO_2_ / FiO_2_	0.83	0.003	(0.74–0.94)

^a^Per 10–point increase in chest radiograph score and PaO_2_/FiO_2_.

## Discussion

The primary objectives of this study was to test the correlation between pulmonary oedema assessed using the single indicator transpulmonary thermodilution EVLWI measurement and a validated chest radiograph score in patients with ALI/ARDS as well as to test their predictive value for mortality. There was only a moderate positive correlation between the chest radiograph findings and the EVLWI measurement. This correlation was lower, but similar to those of prior studies done more than 20 years ago in which the correlation ranged from r = 0.45-0.83 [12-15]. As a dichotomous test, a chest radiograph score >85 was sensitive in determining whether EVLW is present, but a score >85 was not specific for EVLW ≥16 ml/kg.

Using the single-indicator transpulmonary thermodilution method to measure EVLWI is attractive for many reasons. First, this method generates a quantitative measure of EVLW, in contrast to the chest radiograph, which must be interpreted qualitatively by clinicians and is susceptible to interobserver disagreement. We tried to reduce interobserver variability by using an objective validated chest radiograph scoring system [33]. Once ALI/ARDS is established, current practice relies on clinicians accurately interpreting successive chest radiographs to determine whether pulmonary oedema is accumulating or resolving. Although chest radiographs are relatively inexpensive, readily available, and noninvasive, quantitative measures are clinically more useful than qualitative measures. For example, specific cutoffs in a quantitative measure such as EVLWI could be used to guide patient management in a fluid management protocol. Second, the chest radiograph is insensitive to small changes in the quantity of EVLW present [36]. In contrast, small changes (10-20%) in EVLW can be detected using the single indicator method [37]. Detecting small changes in EVLWI may allow for earlier diagnosis of ALI/ARDS and more precise monitoring of responses to therapeutic measures. Finally, lung inflation affects the appearance of the chest radiograph; likewise, atelectasis may be difficult to differentiate from airspace opacities consistent with alveolar oedema [38]. However, these potential benefits of the transpulmonary thermodilution method must be weighed against the potential complications of this invasive method, including the risk of bleeding at the site of vascular access, arterial injury, and thrombosis.

The change in daily chest radiograph scores was not predictive of time to death. In contrast, EVLWI changes over consecutive ICU days predicted mortality. This builds upon our previous data that baseline EVLWI > 16 ml/kg predicts mortality in ALI [32]. Other studies have reported that EVLW may be an independent predictor of mortality for ICU patients [4]. One study reported a mortality rate of 65% when EVLW was >15 ml/kg [4], and another found that EVLWI >16 ml/kg predicted mortality with 100% specificity and 86% sensitivity [3]. In addition, EVLWI may have predictive value during the entire clinical course because maximum EVLWI is a predictor of mortality [3,4]. This is consistent with our finding that the change in EVLWI measurements over the ICU course was predictive of mortality. Taken together, these observations indicate that EVLWI measurements may provide a quantitative assessment of lung injury severity, which could be used to guide therapy in ALI/ARDS. Consistent with this, prior studies have reported that EVLWI measurements can be successfully used to monitor response to β agonist therapy [39,40]. The next step would be to design a clinical trial in which EVLWI measurements are used to guide fluid and diuretic therapy, and within this same trial, determine if decreases in EVLW are associated with a decrease in mortality.

The threshold of 16 ml/kg for the EVLWI has been used to identify ARDS patients with a higher mortality. This level indicates a high amount of pulmonary oedema, although levels between 10 and 15 ml/kg represent progressive interstitial and alveolar oedema [41]. Some investigators have concluded that as many as 50% of patients with a clinical diagnosis of ARDS have a normal EVLWI [42], but more prospective data are needed to assess this issue.

There was a trend towards a lower baseline PaO_2_/FiO_2_ ratio in nonsurvivors, and in logistic regression baseline PaO_2_/FiO_2_ ratio predicted ventilator free days and mortality. In addition, the change in PaO_2_/FiO_2_ ratio over consecutive ICU days predicted time to death. Although not consistent in all studies, baseline PaO_2_/FiO_2_ ratio has been reported in some studies to be lower in nonsurvivors [43-46] and predicts mortality in univariate analyses [43,44,46]. In addition, in one large cohort study, PaO_2_/FiO_2_ ratio was an independent predictor of mortality [44]. However, these studies did not test the change in PaO_2_/FiO_2_ ratio over consecutive ICU days as a predictor of mortality. A limitation of PaO_2_/FiO_2_ ratio as a predictor of outcome is the fact that it can be modified independently by adjustment in ventilatory settings, a limitation to which measurement of EVLWI is not susceptible.

The baseline chest radiograph score differed between survivors and non-survivors, but in contrast to EVLWI and PaO_2_/FiO_2_, did not predict mortality. The change in daily chest radiograph scores did not predict time to death although it did predict ventilator-free days. Most prior studies of chest radiograph findings in patients with pulmonary oedema examined the ability of the chest radiograph to predict the amount of EVLW present, not mortality [12,13,15,17,47]. Our chest radiograph scoring system is easy to apply clinically, has been validated [33], and has a high sensitivity for the detection of EVLW.

Measurement of the EVLWI using the transpulmonary thermodilution method may serve two important purposes. First, measuring EVLWI using this method may be useful to confirm the presence of an increase in pulmonary oedema. In addition, by using the transpulmonary thermodilution method, EVLWI is measured quantitatively. Trending the EVLWI measurements over consecutive ICU days predicts mortality and may be of value for guiding therapy.

Our study has some limitations. First, the total number of deaths (18/59 patients) limited our ability to perform a multivariable regression analysis controlling for potentially confounding conditions that may affect the risk of death. However, because we collected data on multiple days for each patient, we did have sufficient power to detect associations between the chest radiograph score and EVLWI. More importantly, we could use Cox regression models to determine whether the change in each of these predictors (chest radiograph score, EVLWI, and PaO_2_/FiO_2_) over time predicted time to death. Second, when scoring the chest radiographs, we only considered the proportion of the airspace that was affected, and not the density of the alveolar oedema. Certain characteristic signs, such as pulmonary congestion and vascular redistribution, are associated with small increases in EVLW [8]. As the quantity of EVLW increases, densities occupy a greater proportion of the total airspace, and as EVLW further increases, the density of the airspace opacities also worsens [8]. If we had accounted for the density in addition to the proportion of airspace affected, the correlation between the chest radiograph score and EVLWI might have been stronger, and chest radiograph score may have been predictive of mortality. Third, many of the chest radiographs were scored at the maximum chest radiograph score. It would have been ideal to have chest radiograph scores distributed more evenly across the entire scoring range to provide more variability to determine how these scores match with the wide range of EVLWI measurements. Fourth, we did not use the PiCCO device to estimate lung vascular permeability, which other investigators have used [48]. Fifth, we did not quantify pleural effusions by ultrasound or CT [49], and sixth, we did not quantify fluid balance in these patients [50].

## Conclusions

EVLW measured by the chest radiograph only modestly predicted EVLWI as measured by the transpulmonary thermodilution technique. However, unlike the chest radiograph score, transpulmonary thermodilution EVLWI measurements over consecutive days predicted mortality in patients with ALI/ARDS, as others have reported [51,52]. Because it is quantitative and sensitive, and predicts mortality, measurement of EVLWI using single-indicator transpulmonary thermodilution may provide rapidly available and specific information at the bedside in ALI/ARDS. These characteristics suggest that this measurement may be a valuable resource as a prognostic and assessment tool, in both clinical practice and research studies, although prospective studies will be needed to test this hypothesis further.

## Competing interests

None of the authors have a financial relationship with a commercial entity that has an interest in the subject of this manuscript.

## Authors’ contributions

LB, CC, MM and DM designed the study. LB, CC, JH and TC contributed to data collection and chest radiograph interpretations. LB and CC analyzed the data. All authors contributed to interpretation of the study results. LB wrote the first draft of the manuscript. All authors reviewed the manuscript and approved the final draft.
